# Predictors of Readmission to an Acute Psychiatric Inpatient Unit

**DOI:** 10.1192/bjo.2024.518

**Published:** 2024-08-01

**Authors:** Aidan Turkington, Emily Stirling

**Affiliations:** Belfast Health and Social Care Trust, Belfast, United Kingdom

## Abstract

**Aims:**

The Acute Mental Health Inpatient Centre is an 80 bed acute psychiatric inpatient unit in Belfast. The inpatient unit is frequently over capacity resulting in the use of contingency beds or delays in accessing acute inpatient care. Readmissions to hospital after discharge remain a challenge for the service. A service evaluation project was designed to quantify the number of patients being readmitted and determine demographic and diagnostic variables associated with risk of readmission.

**Methods:**

1084 sequential discharges were examined between Jan 2022 and Feb 2023. Age, gender and length of stay (LOS) were determined.

For each case it was determined whether or not the case was a readmission, defined as having been discharged within the previous three months.

Diagnosis was available on 1017 (94%) cases and was categorized as schizophrenia/non-affective psychosis, bipolar affective disorder, non-psychotic mental illness, personality disorder, adjustment disorder, substance misuse disorder and dementia/cognitive impairment.

Social deprivation status was determined for each case based on the address of admission and using social deprivation data from the Northern Ireland census, 2017.

Outcome of discharge was readmission at one week, one month and three months.

**Results:**

For the entire cohort, readmission rates at one week, one month and three months were 5.1%, 13.6% and 20.7% respectively.

Risk of readmission was significantly increased in cases with a diagnosis of personality disorder, a LOS under two weeks and female gender.

Individuals who had been readmitted to hospital within three months of the index admission were significantly more likely to be readmitted in the subsequent three months.

Data on social deprivation is currently undergoing analysis and will be available in due course.

Logistic regression was performed to determine how the variables impacted on risk of readmission at 3 months. In the final model, diagnosis of personality disorder (OR 3.1; 95% CI 2.0, 4.7; p < 0.001), diagnosis of schizophrenia (OR 1.8; 95% CI 1.1, 2.7; p < 0.01), the admission being a readmission (OR 3.4; 95% CI 2.4, 5.0; p < 0.001), a LOS less than 2 weeks (OR 1.9; 95% CI 1.3, 2.7; p < 0.001) and female gender (OR 1.7; 95% CI 1.2, 2.4; p < 0.01) all predicted readmission within three months.

**Conclusion:**

The service evaluation project has allowed individuals at a higher risk of readmission to be identified. This study has informed local strategies now being implemented to target community care and provide timely interventions to those groups at highest risk of readmission.

